# (*E*)-2-[2-(2-Nitro­phen­yl)ethen­yl]-8-quinolyl acetate

**DOI:** 10.1107/S1600536809044158

**Published:** 2009-10-31

**Authors:** Li-Yan Zhang, Yan-Ping Huo

**Affiliations:** a Department of Chemistry, Huangshan University, Huangshan 245041, People’s Republic of China; b Faculty of Chemical Engineering and Light Industry, Guangdong University of Technology, Guangdong 510006, People’s Republic of China

## Abstract

The title compound, C_19_H_14_N_2_O_4_, crystallizes with two molecules with very similar conformations in the asymmetric unit; the angles between the two ring systems are 8.7 (1) and 4.2 (1)°. In the crystal, inter­molecular π–π inter­actions [centroid–centroid distance 3.973 (1) Å] lead to a three-dimensional network.

## Related literature

For the biological properties of 8-hydroxy­quinoline derivatives, see: Chen *et al.* (2002 ▶); Fakhfakh *et al.* (2003 ▶); Mekouar *et al.* (1998 ▶); Ouali *et al.* (2000 ▶); Storz *et al.* (2004 ▶); Zeng, Wang *et al.* (2006 ▶). For a related crystal structure, see: Zeng, OuYang *et al.* (2006 ▶).
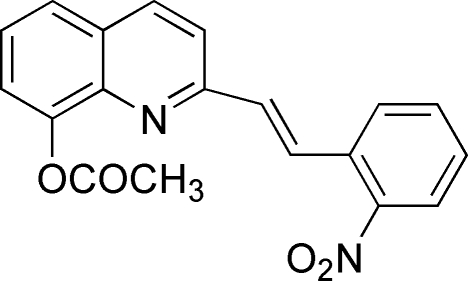


## Experimental

### 

#### Crystal data

C_19_H_14_N_2_O_4_
*M* *_r_* = 334.32
Orthorhombic, 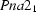

*a* = 25.8466 (13) Å
*b* = 11.8451 (6) Å
*c* = 10.5870 (5) Å
*V* = 3241.3 (3) Å^3^
*Z* = 8
Mo *K*α radiation
μ = 0.10 mm^−1^
*T* = 173 K
0.47 × 0.45 × 0.26 mm

#### Data collection

Bruker SMART 1000 CCD diffractometer
Absorption correction: multi-scan (*SADABS*; Bruker, 2001 ▶) *T*_min_ = 0.956, *T*_max_ = 0.975
17451 measured reflections
3731 independent reflections
2785 reflections with *I* > 2σ(*I*)
*R*_int_ = 0.039

#### Refinement

*R*[*F* ^2^ > 2σ(*F* ^2^)] = 0.058
*wR*(*F* ^2^) = 0.171
*S* = 1.04
3731 reflections
453 parameters
7 restraints
H-atom parameters constrained
Δρ_max_ = 0.56 e Å^−3^
Δρ_min_ = −0.22 e Å^−3^

### 

Data collection: *SMART* (Bruker, 2001 ▶); cell refinement: *SAINT-Plus* (Bruker, 2003 ▶); data reduction: *SAINT-Plus*; program(s) used to solve structure: *SHELXTL* (Sheldrick, 2008 ▶); program(s) used to refine structure: *SHELXTL*; molecular graphics: *SHELXTL*; software used to prepare material for publication: *SHELXTL*.

## Supplementary Material

Crystal structure: contains datablocks I, global. DOI: 10.1107/S1600536809044158/wn2356sup1.cif

Structure factors: contains datablocks I. DOI: 10.1107/S1600536809044158/wn2356Isup2.hkl

Additional supplementary materials:  crystallographic information; 3D view; checkCIF report

## Figures and Tables

**Fig. 1.**
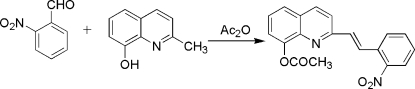
The synthesis of (E)-2-[2-(2-nitrophenyl)ethenyl]-8-acetoxyquinoline.

**Fig. 2.**
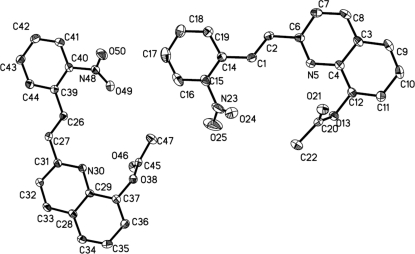
View of the asymmetric unit of the title compound. Hydrogen atoms have been omitted.

**Fig. 3.**
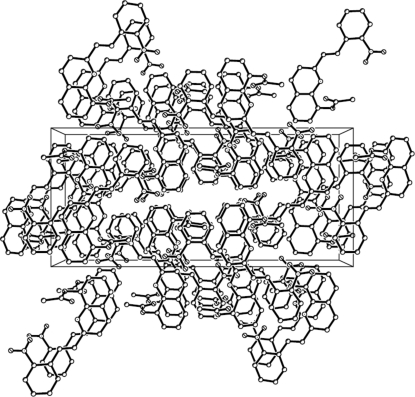
View of the three-dimensional supramolecular structure.

## supplementary crystallographic information

### Comment

8-Hydroxyquinoline derivatives are important constituents in a variety of classes of pharmaceutically important compounds. They have generated interest as a new class of potent HIV-1 integrase inhibitors (Mekouar *et al.*, 1998), for modeling of the inhibition of retroviral integrases (Ouali *et al.*, 2000), as protein tyrosine kinase inhibitors (Chen *et al.*, 2002), as protozoal and retroviral co-infections (Fakhfakh *et al.*, 2003), and as anti-HIV-1 agents (Storz *et al.*, 2004). Zeng, Wang *et al.* (2006) reported that a series of 8-hydroxyquinoline derivatives with vinyl substituents at the 2-position could induce the proliferation of rMSCs (rat mesenchymal stem cells). With these findings, some analogs need to be synthesized for structure activity relationship research to find more potent molecules.

One of these analogs, the title compound, (*E*)-2-[2-(2-nitrophenyl)ethenyl]-8-acetoxyquinoline, was prepared by the Knoevenagel condensation reaction between 8-hydroxyquinaldine and 2-nitrobenzaldehyde (Zeng, OuYang *et al.* (2006)) (Fig. 1). To provide structural information for the title compound, we studied its crystal structure.

The molecular structure of the two molecules per asymmetric unit is illustrated in Fig. 2. In one molecule, the angle between the two ring systems is 8.7 (1)°; in the other it is 4.2 (1)°. In this crystal structure, the ethylenic bond lengths for C1—C2 and C26—C27 are 1.322 (5) and 1.329 (5) Å, respectively. The analogous bond reported by Zeng, OuYang *et al.* (2006) has a length of 1.335 (2) Å. Intermolecular π–π interactions, with a centroid···centroid distance of 3.973 (1) Å, lead directly to a three-dimensional supramolecular network (Fig. 3).

### Experimental

To a solution of 8-hydroxyquinaldine (1.19 g, 7.5 mmol) in acetic anhydride (5 ml) was added 2-nitrobenzaldehyde (1.12 g, 7.5 mmol). The mixture was heated under reflux for 11 h. After cooling, it was poured into ice water (50 ml) and stirred overnight. The yellow solid obtained was filtered and washed with water. The solid residue was recrystallized from CH_2_Cl_2 _to afford the title compound (2.04 g, 75%). mp 143–144 °C. 20 mg was dissolved in 10 ml (EtOAc:petroleum ether 1:4) and the solution was kept at room temperature for 4 d. Natural evaporation gave orange single crystals suitable for X-ray analysis.

### Refinement

All H atoms were positioned geometrically and refined using a riding model (including free rotation about the acetoxy C—C bond), with C—H = 0.95 Å (Csp^2^) and 0.98 Å (methyl C); *U*_iso_(H) = k*U*_eq_(C), where k = 1.5 for methyl H atoms and 1.2 for all other H atoms. In the absence of significant anomalous scattering effects, Friedel pairs were merged.

### Crystal data

**Table:**

C_19_H_14_N_2_O_4_	*F*(000) = 1392
*M**_r_* = 334.32	*D*_x_ = 1.370 Mg m^−^^3^
Orthorhombic, *P**n**a*2_1_	Mo *K*α radiation, λ = 0.71073 Å
Hall symbol: P 2c -2n	Cell parameters from 5386 reflections
*a* = 25.8466 (13) Å	θ = 2.3–26.3°
*b* = 11.8451 (6) Å	µ = 0.10 mm^−^^1^
*c* = 10.5870 (5) Å	*T* = 173 K
*V* = 3241.3 (3) Å^3^	Block, yellow
*Z* = 8	0.47 × 0.45 × 0.26 mm

### Data collection

**Table:**

Bruker SMART 1000 CCD diffractometer	3731 independent reflections
Radiation source: fine-focus sealed tube	2785 reflections with *I* > 2σ(*I*)
graphite	*R*_int_ = 0.039
ω scans	θ_max_ = 27.1°, θ_min_ = 1.6°
Absorption correction: multi-scan (*SADABS*; Bruker, 2001)	*h* = −29→33
*T*_min_ = 0.956, *T*_max_ = 0.975	*k* = −13→15
17451 measured reflections	*l* = −13→13

### Refinement

**Table:**

Refinement on *F*^2^	Primary atom site location: structure-invariant direct methods
Least-squares matrix: full	Secondary atom site location: difference Fourier map
*R*[*F*^2^ > 2σ(*F*^2^)] = 0.058	Hydrogen site location: inferred from neighbouring sites
*wR*(*F*^2^) = 0.171	H-atom parameters constrained
*S* = 1.04	*w* = 1/[σ^2^(*F*_o_^2^) + (0.1126*P*)^2^ + 0.5654*P*] where *P* = (*F*_o_^2^ + 2*F*_c_^2^)/3
3731 reflections	(Δ/σ)_max_ = 0.004
453 parameters	Δρ_max_ = 0.56 e Å^−^^3^
7 restraints	Δρ_min_ = −0.22 e Å^−^^3^

### Special details

**Table:**

Experimental. ^1^H NMR (CDCl_3_, 300 MHz) δ 8.17–8.26 (m, 2H), 7.94 (d, J=7.5 Hz, 1H), 7.82 (d, J=7.8 Hz 1H), 7.64–7.70 (m, 4H), 7.47–7.53 (m, 3H), 2.58(s, 3H); IR (KBr, cm^-1^): 3067, 1723, 1577, 1520, 1457, 1175, 1128, 970, 850, 765, 708; ESI-MS m/*z*: 335.9 ([*M*+H]^+^); Elemental analysis: found C: 68.45, H: 4.40, N: 8.38 (%)
Geometry. All e.s.d.'s (except the e.s.d. in the dihedral angle between two l.s. planes) are estimated using the full covariance matrix. The cell e.s.d.'s are taken into account individually in the estimation of e.s.d.'s in distances, angles and torsion angles; correlations between e.s.d.'s in cell parameters are only used when they are defined by crystal symmetry. An approximate (isotropic) treatment of cell e.s.d.'s is used for estimating e.s.d.'s involving l.s. planes.
Refinement. Refinement of *F*^2^ against ALL reflections. The weighted *R*-factor *wR* and goodness of fit *S* are based on *F*^2^, conventional *R*-factors *R* are based on *F*, with *F* set to zero for negative *F*^2^. The threshold expression of *F*^2^ > σ(*F*^2^) is used only for calculating *R*-factors(gt) *etc*. and is not relevant to the choice of reflections for refinement. *R*-factors based on *F*^2^ are statistically about twice as large as those based on *F*, and *R*- factors based on ALL data will be even larger.

### Fractional atomic coordinates and isotropic or equivalent isotropic displacement parameters (Å^2^)

**Table:**

	*x*	*y*	*z*	*U*_iso_*/*U*_eq_	
C1	0.79027 (14)	0.4406 (3)	0.2939 (4)	0.0354 (9)	
H1	0.7753	0.5118	0.2748	0.043*	
C2	0.83678 (14)	0.4416 (3)	0.3458 (4)	0.0347 (8)	
H2	0.8522	0.3712	0.3664	0.042*	
C3	0.92068 (14)	0.7392 (3)	0.4307 (4)	0.0337 (9)	
C4	0.87102 (14)	0.7393 (3)	0.3743 (4)	0.0301 (8)	
N5	0.84409 (11)	0.6440 (3)	0.3460 (3)	0.0299 (7)	
C6	0.86607 (13)	0.5452 (3)	0.3734 (4)	0.0321 (8)	
C7	0.91645 (16)	0.5376 (4)	0.4257 (4)	0.0383 (9)	
H7	0.9315	0.4656	0.4403	0.046*	
C8	0.94344 (14)	0.6324 (4)	0.4549 (4)	0.0396 (10)	
H8	0.9770	0.6273	0.4910	0.048*	
C9	0.94489 (16)	0.8434 (4)	0.4613 (4)	0.0432 (10)	
H9	0.9779	0.8436	0.5007	0.052*	
C10	0.92101 (17)	0.9425 (4)	0.4346 (4)	0.0447 (10)	
H10	0.9372	1.0116	0.4569	0.054*	
C11	0.87218 (16)	0.9444 (3)	0.3739 (4)	0.0393 (9)	
H11	0.8560	1.0143	0.3544	0.047*	
C12	0.84871 (14)	0.8454 (3)	0.3436 (4)	0.0310 (8)	
O13	0.79975 (10)	0.8466 (2)	0.2896 (3)	0.0343 (6)	
C14	0.75968 (15)	0.3408 (3)	0.2631 (4)	0.0360 (9)	
C15	0.71059 (16)	0.3464 (4)	0.2113 (5)	0.0470 (11)	
C16	0.67993 (18)	0.2511 (5)	0.1900 (6)	0.0594 (15)	
H16	0.6458	0.2589	0.1576	0.071*	
C17	0.6995 (2)	0.1471 (5)	0.2162 (5)	0.0580 (14)	
H17	0.6794	0.0815	0.2005	0.070*	
C18	0.74860 (19)	0.1373 (4)	0.2656 (5)	0.0492 (11)	
H18	0.7623	0.0646	0.2834	0.059*	
C19	0.77801 (16)	0.2319 (3)	0.2896 (4)	0.0395 (10)	
H19	0.8116	0.2231	0.3249	0.047*	
C20	0.79586 (15)	0.8136 (3)	0.1658 (4)	0.0330 (8)	
O21	0.83315 (11)	0.7982 (2)	0.1009 (3)	0.0417 (7)	
C22	0.74072 (15)	0.8026 (4)	0.1270 (5)	0.0473 (11)	
H22A	0.7385	0.8011	0.0346	0.071*	
H22B	0.7210	0.8670	0.1594	0.071*	
H22C	0.7264	0.7324	0.1614	0.071*	
N23	0.68857 (17)	0.4577 (5)	0.1744 (6)	0.0746 (17)	
O24	0.7137 (2)	0.5202 (4)	0.1073 (7)	0.101 (2)	
O25	0.64504 (18)	0.4750 (5)	0.2109 (7)	0.124 (2)	
C26	0.46521 (13)	−0.0779 (3)	0.1266 (4)	0.0304 (8)	
H26	0.4808	−0.0079	0.1483	0.036*	
C27	0.41923 (15)	−0.0756 (3)	0.0705 (4)	0.0358 (9)	
H27	0.4045	−0.1458	0.0459	0.043*	
C28	0.33041 (14)	0.2153 (3)	−0.0099 (4)	0.0321 (8)	
C29	0.37963 (13)	0.2194 (3)	0.0491 (4)	0.0290 (8)	
N30	0.40900 (11)	0.1266 (3)	0.0752 (3)	0.0308 (7)	
C31	0.38950 (14)	0.0269 (3)	0.0438 (4)	0.0318 (8)	
C32	0.33986 (15)	0.0140 (3)	−0.0117 (4)	0.0378 (9)	
H32	0.3268	−0.0592	−0.0298	0.045*	
C33	0.31110 (15)	0.1068 (4)	−0.0387 (4)	0.0376 (9)	
H33	0.2780	0.0988	−0.0768	0.045*	
C34	0.30335 (15)	0.3162 (3)	−0.0367 (4)	0.0364 (9)	
H34	0.2705	0.3132	−0.0767	0.044*	
C35	0.32446 (16)	0.4174 (4)	−0.0053 (4)	0.0415 (10)	
H35	0.3063	0.4849	−0.0247	0.050*	
C36	0.37324 (15)	0.4241 (3)	0.0561 (4)	0.0383 (9)	
H36	0.3876	0.4955	0.0772	0.046*	
C37	0.39908 (14)	0.3276 (3)	0.0842 (4)	0.0314 (8)	
O38	0.44801 (10)	0.3328 (2)	0.1386 (3)	0.0339 (6)	
C39	0.49328 (13)	−0.1817 (3)	0.1570 (4)	0.0305 (8)	
C40	0.54472 (14)	−0.1848 (3)	0.2014 (4)	0.0340 (9)	
C41	0.57112 (16)	−0.2840 (4)	0.2254 (5)	0.0425 (11)	
H41	0.6063	−0.2822	0.2513	0.051*	
C42	0.54573 (16)	−0.3852 (4)	0.2111 (5)	0.0458 (10)	
H42	0.5628	−0.4540	0.2311	0.055*	
C43	0.49544 (15)	−0.3867 (3)	0.1678 (5)	0.0428 (10)	
H43	0.4782	−0.4568	0.1564	0.051*	
C44	0.46994 (14)	−0.2877 (3)	0.1411 (4)	0.0360 (9)	
H44	0.4354	−0.2912	0.1109	0.043*	
C45	0.45270 (15)	0.2938 (3)	0.2601 (4)	0.0345 (9)	
O46	0.41633 (11)	0.2756 (3)	0.3261 (3)	0.0436 (7)	
C47	0.50824 (17)	0.2795 (4)	0.2951 (5)	0.0510 (12)	
H47A	0.5112	0.2715	0.3870	0.076*	
H47B	0.5279	0.3458	0.2674	0.076*	
H47C	0.5221	0.2118	0.2540	0.076*	
N48	0.57481 (13)	−0.0810 (3)	0.2185 (4)	0.0428 (9)	
O49	0.55389 (14)	0.0009 (3)	0.2665 (4)	0.0621 (10)	
O50	0.62029 (12)	−0.0830 (3)	0.1858 (4)	0.0628 (10)	

### Atomic displacement parameters (Å^2^)

**Table:**

	*U*^11^	*U*^22^	*U*^33^	*U*^12^	*U*^13^	*U*^23^
C1	0.037 (2)	0.033 (2)	0.036 (2)	0.0056 (15)	−0.0016 (16)	−0.0013 (16)
C2	0.0349 (19)	0.0296 (19)	0.039 (2)	0.0053 (15)	0.0015 (16)	0.0038 (16)
C3	0.0286 (19)	0.049 (2)	0.023 (2)	−0.0057 (16)	−0.0027 (16)	0.0050 (16)
C4	0.0296 (19)	0.038 (2)	0.0223 (19)	−0.0017 (15)	0.0006 (15)	0.0022 (15)
N5	0.0253 (15)	0.0350 (17)	0.0293 (17)	0.0025 (12)	−0.0008 (13)	0.0008 (13)
C6	0.0279 (18)	0.036 (2)	0.032 (2)	0.0030 (15)	0.0030 (15)	0.0058 (16)
C7	0.039 (2)	0.045 (2)	0.031 (2)	0.0094 (17)	−0.0005 (17)	0.0089 (18)
C8	0.0250 (19)	0.059 (3)	0.035 (2)	0.0009 (17)	−0.0046 (16)	0.0100 (19)
C9	0.031 (2)	0.061 (3)	0.038 (2)	−0.0135 (19)	−0.0060 (18)	0.002 (2)
C10	0.048 (2)	0.049 (3)	0.037 (2)	−0.021 (2)	−0.0052 (19)	−0.0014 (19)
C11	0.040 (2)	0.037 (2)	0.040 (2)	−0.0045 (17)	−0.0010 (18)	−0.0002 (18)
C12	0.0263 (18)	0.038 (2)	0.029 (2)	−0.0030 (14)	0.0012 (15)	−0.0016 (15)
O13	0.0299 (13)	0.0375 (15)	0.0356 (15)	0.0027 (10)	−0.0024 (11)	−0.0036 (11)
C14	0.032 (2)	0.039 (2)	0.036 (2)	0.0017 (16)	0.0026 (16)	−0.0066 (17)
C15	0.039 (2)	0.060 (3)	0.042 (3)	0.0037 (19)	−0.004 (2)	−0.016 (2)
C16	0.036 (2)	0.089 (4)	0.054 (3)	−0.007 (2)	−0.006 (2)	−0.023 (3)
C17	0.062 (3)	0.067 (4)	0.046 (3)	−0.027 (3)	0.012 (2)	−0.008 (2)
C18	0.058 (3)	0.042 (2)	0.047 (3)	−0.011 (2)	0.010 (2)	0.001 (2)
C19	0.040 (2)	0.038 (2)	0.041 (3)	−0.0021 (17)	0.0066 (19)	0.0015 (18)
C20	0.0354 (19)	0.0249 (18)	0.039 (2)	0.0032 (14)	−0.0032 (17)	0.0015 (16)
O21	0.0397 (16)	0.0473 (16)	0.0382 (17)	0.0054 (12)	0.0026 (13)	0.0020 (13)
C22	0.036 (2)	0.061 (3)	0.044 (3)	0.0046 (19)	−0.0094 (19)	0.000 (2)
N23	0.045 (3)	0.089 (4)	0.090 (4)	0.028 (2)	−0.035 (3)	−0.039 (3)
O24	0.102 (4)	0.053 (2)	0.150 (5)	0.014 (2)	−0.063 (4)	−0.004 (3)
O25	0.077 (3)	0.167 (5)	0.126 (4)	0.071 (3)	−0.028 (3)	−0.049 (4)
C26	0.0292 (18)	0.0292 (18)	0.0326 (19)	−0.0033 (14)	0.0012 (15)	0.0004 (15)
C27	0.038 (2)	0.0288 (19)	0.041 (2)	0.0020 (15)	−0.0058 (17)	−0.0073 (16)
C28	0.0293 (19)	0.043 (2)	0.024 (2)	0.0012 (16)	−0.0011 (15)	−0.0016 (16)
C29	0.0227 (17)	0.036 (2)	0.028 (2)	0.0018 (14)	0.0017 (15)	0.0010 (15)
N30	0.0277 (15)	0.0323 (16)	0.0324 (18)	0.0018 (12)	−0.0006 (13)	−0.0036 (13)
C31	0.0308 (18)	0.035 (2)	0.029 (2)	−0.0003 (15)	−0.0018 (15)	−0.0031 (15)
C32	0.036 (2)	0.035 (2)	0.043 (2)	−0.0014 (16)	−0.0104 (17)	−0.0033 (17)
C33	0.0266 (18)	0.047 (2)	0.039 (2)	−0.0024 (16)	−0.0088 (16)	−0.0033 (19)
C34	0.031 (2)	0.043 (2)	0.035 (2)	0.0056 (16)	−0.0038 (17)	0.0001 (18)
C35	0.039 (2)	0.043 (2)	0.042 (3)	0.0125 (18)	0.0008 (18)	0.0050 (19)
C36	0.039 (2)	0.033 (2)	0.043 (2)	0.0005 (16)	0.0005 (18)	−0.0004 (18)
C37	0.0279 (18)	0.038 (2)	0.029 (2)	−0.0001 (14)	0.0028 (15)	−0.0016 (16)
O38	0.0295 (13)	0.0325 (13)	0.0398 (16)	−0.0051 (10)	−0.0011 (11)	0.0029 (12)
C39	0.0291 (18)	0.0318 (18)	0.031 (2)	−0.0006 (14)	0.0026 (15)	−0.0011 (16)
C40	0.0313 (19)	0.036 (2)	0.035 (2)	−0.0040 (15)	−0.0038 (17)	−0.0019 (17)
C41	0.032 (2)	0.045 (3)	0.051 (3)	0.0033 (17)	−0.006 (2)	0.000 (2)
C42	0.043 (2)	0.037 (2)	0.058 (3)	0.0068 (17)	0.000 (2)	0.000 (2)
C43	0.035 (2)	0.032 (2)	0.060 (3)	−0.0005 (16)	0.0018 (19)	−0.0030 (19)
C44	0.0267 (18)	0.039 (2)	0.042 (2)	−0.0043 (15)	0.0031 (16)	−0.0013 (18)
C45	0.038 (2)	0.0258 (18)	0.040 (2)	−0.0047 (15)	−0.0043 (17)	−0.0021 (16)
O46	0.0421 (17)	0.0522 (18)	0.0366 (17)	−0.0094 (13)	0.0040 (14)	−0.0005 (14)
C47	0.043 (3)	0.066 (3)	0.043 (3)	−0.004 (2)	−0.010 (2)	0.008 (2)
N48	0.045 (2)	0.0363 (19)	0.047 (2)	−0.0037 (15)	−0.0175 (17)	0.0026 (16)
O49	0.065 (2)	0.0429 (19)	0.079 (3)	−0.0009 (16)	−0.0301 (19)	−0.0133 (18)
O50	0.0353 (16)	0.062 (2)	0.091 (3)	−0.0134 (15)	−0.0157 (17)	0.009 (2)

### Geometric parameters (Å, °)

**Table:**

C1—C2	1.322 (5)	C26—C27	1.329 (5)
C1—C14	1.459 (5)	C26—C39	1.464 (5)
C1—H1	0.9500	C26—H26	0.9500
C2—C6	1.471 (5)	C27—C31	1.465 (5)
C2—H2	0.9500	C27—H27	0.9500
C3—C4	1.415 (5)	C28—C33	1.412 (6)
C3—C8	1.418 (6)	C28—C34	1.414 (5)
C3—C9	1.421 (6)	C28—C29	1.418 (5)
C4—N5	1.361 (5)	C29—N30	1.364 (5)
C4—C12	1.420 (5)	C29—C37	1.426 (5)
N5—C6	1.332 (5)	N30—C31	1.327 (5)
C6—C7	1.418 (5)	C31—C32	1.419 (5)
C7—C8	1.358 (6)	C32—C33	1.358 (6)
C7—H7	0.9500	C32—H32	0.9500
C8—H8	0.9500	C33—H33	0.9500
C9—C10	1.357 (6)	C34—C35	1.358 (6)
C9—H9	0.9500	C34—H34	0.9500
C10—C11	1.417 (6)	C35—C36	1.420 (6)
C10—H10	0.9500	C35—H35	0.9500
C11—C12	1.358 (5)	C36—C37	1.357 (5)
C11—H11	0.9500	C36—H36	0.9500
C12—O13	1.389 (4)	C37—O38	1.391 (5)
O13—C20	1.372 (5)	O38—C45	1.372 (5)
C14—C15	1.384 (6)	C39—C44	1.403 (5)
C14—C19	1.403 (6)	C39—C40	1.411 (5)
C15—C16	1.397 (7)	C40—C41	1.382 (6)
C15—N23	1.488 (7)	C40—N48	1.466 (5)
C16—C17	1.361 (8)	C41—C42	1.375 (6)
C16—H16	0.9500	C41—H41	0.9500
C17—C18	1.377 (7)	C42—C43	1.378 (6)
C17—H17	0.9500	C42—H42	0.9500
C18—C19	1.378 (6)	C43—C44	1.375 (6)
C18—H18	0.9500	C43—H43	0.9500
C19—H19	0.9500	C44—H44	0.9500
C20—O21	1.197 (5)	C45—O46	1.191 (5)
C20—C22	1.489 (5)	C45—C47	1.492 (6)
C22—H22A	0.9800	C47—H47A	0.9800
C22—H22B	0.9800	C47—H47B	0.9800
C22—H22C	0.9800	C47—H47C	0.9800
N23—O25	1.207 (6)	N48—O49	1.222 (5)
N23—O24	1.215 (7)	N48—O50	1.226 (5)
			
C2—C1—C14	126.4 (4)	C27—C26—C39	123.9 (3)
C2—C1—H1	116.8	C27—C26—H26	118.0
C14—C1—H1	116.8	C39—C26—H26	118.0
C1—C2—C6	123.9 (3)	C26—C27—C31	124.9 (3)
C1—C2—H2	118.0	C26—C27—H27	117.6
C6—C2—H2	118.0	C31—C27—H27	117.6
C4—C3—C8	117.0 (4)	C33—C28—C34	123.4 (3)
C4—C3—C9	119.7 (4)	C33—C28—C29	116.3 (3)
C8—C3—C9	123.4 (4)	C34—C28—C29	120.2 (3)
N5—C4—C3	123.8 (3)	N30—C29—C28	124.1 (3)
N5—C4—C12	118.4 (3)	N30—C29—C37	118.4 (3)
C3—C4—C12	117.8 (3)	C28—C29—C37	117.5 (3)
C6—N5—C4	117.6 (3)	C31—N30—C29	117.1 (3)
N5—C6—C7	122.2 (4)	N30—C31—C32	122.9 (3)
N5—C6—C2	118.0 (3)	N30—C31—C27	119.4 (3)
C7—C6—C2	119.8 (3)	C32—C31—C27	117.7 (3)
C8—C7—C6	120.6 (4)	C33—C32—C31	119.7 (4)
C8—C7—H7	119.7	C33—C32—H32	120.2
C6—C7—H7	119.7	C31—C32—H32	120.2
C7—C8—C3	118.9 (4)	C32—C33—C28	119.9 (3)
C7—C8—H8	120.6	C32—C33—H33	120.1
C3—C8—H8	120.6	C28—C33—H33	120.1
C10—C9—C3	120.2 (4)	C35—C34—C28	119.9 (4)
C10—C9—H9	119.9	C35—C34—H34	120.1
C3—C9—H9	119.9	C28—C34—H34	120.1
C9—C10—C11	120.9 (4)	C34—C35—C36	121.2 (4)
C9—C10—H10	119.5	C34—C35—H35	119.4
C11—C10—H10	119.5	C36—C35—H35	119.4
C12—C11—C10	119.4 (4)	C37—C36—C35	119.3 (4)
C12—C11—H11	120.3	C37—C36—H36	120.3
C10—C11—H11	120.3	C35—C36—H36	120.3
C11—C12—O13	119.7 (3)	C36—C37—O38	120.1 (3)
C11—C12—C4	121.9 (4)	C36—C37—C29	121.8 (3)
O13—C12—C4	118.3 (3)	O38—C37—C29	117.9 (3)
C20—O13—C12	117.2 (3)	C45—O38—C37	117.0 (3)
C15—C14—C19	115.6 (4)	C44—C39—C40	114.9 (3)
C15—C14—C1	123.2 (4)	C44—C39—C26	120.8 (3)
C19—C14—C1	121.2 (4)	C40—C39—C26	124.2 (3)
C14—C15—C16	123.1 (5)	C41—C40—C39	123.3 (4)
C14—C15—N23	119.8 (4)	C41—C40—N48	115.3 (3)
C16—C15—N23	117.2 (4)	C39—C40—N48	121.3 (3)
C17—C16—C15	119.2 (4)	C42—C41—C40	119.0 (4)
C17—C16—H16	120.4	C42—C41—H41	120.5
C15—C16—H16	120.4	C40—C41—H41	120.5
C16—C17—C18	119.8 (4)	C41—C42—C43	119.8 (4)
C16—C17—H17	120.1	C41—C42—H42	120.1
C18—C17—H17	120.1	C43—C42—H42	120.1
C17—C18—C19	120.6 (5)	C44—C43—C42	120.7 (4)
C17—C18—H18	119.7	C44—C43—H43	119.7
C19—C18—H18	119.7	C42—C43—H43	119.7
C18—C19—C14	121.7 (4)	C43—C44—C39	122.2 (4)
C18—C19—H19	119.2	C43—C44—H44	118.9
C14—C19—H19	119.2	C39—C44—H44	118.9
O21—C20—O13	122.2 (4)	O46—C45—O38	122.7 (4)
O21—C20—C22	126.8 (4)	O46—C45—C47	126.4 (4)
O13—C20—C22	111.0 (3)	O38—C45—C47	110.9 (4)
C20—C22—H22A	109.5	C45—C47—H47A	109.5
C20—C22—H22B	109.5	C45—C47—H47B	109.5
H22A—C22—H22B	109.5	H47A—C47—H47B	109.5
C20—C22—H22C	109.5	C45—C47—H47C	109.5
H22A—C22—H22C	109.5	H47A—C47—H47C	109.5
H22B—C22—H22C	109.5	H47B—C47—H47C	109.5
O25—N23—O24	125.6 (7)	O49—N48—O50	123.8 (4)
O25—N23—C15	115.0 (7)	O49—N48—C40	118.9 (4)
O24—N23—C15	119.3 (4)	O50—N48—C40	117.3 (4)
			
C14—C1—C2—C6	179.3 (4)	C39—C26—C27—C31	177.4 (4)
C8—C3—C4—N5	1.8 (6)	C33—C28—C29—N30	2.0 (6)
C9—C3—C4—N5	−177.5 (4)	C34—C28—C29—N30	−178.1 (4)
C8—C3—C4—C12	−176.9 (4)	C33—C28—C29—C37	−177.3 (4)
C9—C3—C4—C12	3.8 (6)	C34—C28—C29—C37	2.6 (5)
C3—C4—N5—C6	0.1 (6)	C28—C29—N30—C31	−0.7 (6)
C12—C4—N5—C6	178.8 (3)	C37—C29—N30—C31	178.7 (4)
C4—N5—C6—C7	−2.5 (6)	C29—N30—C31—C32	−1.6 (6)
C4—N5—C6—C2	178.7 (3)	C29—N30—C31—C27	−179.5 (3)
C1—C2—C6—N5	0.9 (6)	C26—C27—C31—N30	0.9 (7)
C1—C2—C6—C7	−178.0 (4)	C26—C27—C31—C32	−177.2 (4)
N5—C6—C7—C8	2.9 (6)	N30—C31—C32—C33	2.3 (6)
C2—C6—C7—C8	−178.2 (4)	C27—C31—C32—C33	−179.7 (4)
C6—C7—C8—C3	−0.9 (6)	C31—C32—C33—C28	−0.8 (7)
C4—C3—C8—C7	−1.3 (6)	C34—C28—C33—C32	178.9 (4)
C9—C3—C8—C7	178.0 (4)	C29—C28—C33—C32	−1.2 (6)
C4—C3—C9—C10	−1.2 (7)	C33—C28—C34—C35	179.6 (4)
C8—C3—C9—C10	179.5 (4)	C29—C28—C34—C35	−0.4 (6)
C3—C9—C10—C11	−1.2 (7)	C28—C34—C35—C36	−0.9 (6)
C9—C10—C11—C12	0.9 (7)	C34—C35—C36—C37	−0.3 (6)
C10—C11—C12—O13	176.9 (4)	C35—C36—C37—O38	176.8 (4)
C10—C11—C12—C4	1.8 (6)	C35—C36—C37—C29	2.7 (6)
N5—C4—C12—C11	177.1 (4)	N30—C29—C37—C36	176.8 (4)
C3—C4—C12—C11	−4.1 (6)	C28—C29—C37—C36	−3.8 (6)
N5—C4—C12—O13	2.0 (6)	N30—C29—C37—O38	2.5 (5)
C3—C4—C12—O13	−179.3 (3)	C28—C29—C37—O38	−178.1 (3)
C11—C12—O13—C20	111.1 (4)	C36—C37—O38—C45	113.4 (4)
C4—C12—O13—C20	−73.7 (4)	C29—C37—O38—C45	−72.2 (4)
C2—C1—C14—C15	178.5 (4)	C27—C26—C39—C44	−7.5 (6)
C2—C1—C14—C19	1.1 (7)	C27—C26—C39—C40	171.5 (4)
C19—C14—C15—C16	2.1 (7)	C44—C39—C40—C41	1.3 (6)
C1—C14—C15—C16	−175.4 (4)	C26—C39—C40—C41	−177.7 (4)
C19—C14—C15—N23	−177.3 (4)	C44—C39—C40—N48	177.9 (4)
C1—C14—C15—N23	5.2 (7)	C26—C39—C40—N48	−1.2 (6)
C14—C15—C16—C17	−2.7 (8)	C39—C40—C41—C42	−3.1 (7)
N23—C15—C16—C17	176.7 (5)	N48—C40—C41—C42	−179.8 (4)
C15—C16—C17—C18	1.4 (8)	C40—C41—C42—C43	3.0 (8)
C16—C17—C18—C19	0.4 (8)	C41—C42—C43—C44	−1.3 (8)
C17—C18—C19—C14	−1.0 (7)	C42—C43—C44—C39	−0.4 (7)
C15—C14—C19—C18	−0.3 (7)	C40—C39—C44—C43	0.4 (6)
C1—C14—C19—C18	177.3 (4)	C26—C39—C44—C43	179.5 (4)
C12—O13—C20—O21	−9.5 (5)	C37—O38—C45—O46	−13.8 (5)
C12—O13—C20—C22	171.4 (3)	C37—O38—C45—C47	166.9 (3)
C14—C15—N23—O25	−132.0 (5)	C41—C40—N48—O49	−140.5 (4)
C16—C15—N23—O25	48.6 (7)	C39—C40—N48—O49	42.7 (6)
C14—C15—N23—O24	51.4 (7)	C41—C40—N48—O50	37.9 (6)
C16—C15—N23—O24	−128.0 (6)	C39—C40—N48—O50	−138.9 (4)
